# The relation between emotional intelligence and dark triad traits, limitations of research

**DOI:** 10.1192/j.eurpsy.2025.1934

**Published:** 2025-08-26

**Authors:** M. Valeriia

**Affiliations:** 1 General, child, forensic psychiatry and narcology, P.L. Shupyk National Medical Academy of Postgraduate Education, Kyiv, Ukraine

## Abstract

**Introduction:**

The war has affected every Ukrainian to some extent. With the mobilization comes the increased risk of people with dark triad traits (DT) (psychopathy, machiavellianism and narcissism) to be exposed to stress on the battlefield. Being vulnerable, these individuals are in need of protection for their mental health. One of such protections is the emotional intelligence (EI). This makes the relation between DT and EI highly valued as the basis for future development of effective measures against stress disorders for combatants.

**Objectives:**

The aim of this research is to study the existing knowledge exploring the relation between the emotional intelligence and dark triad traits and to determine the limitations of these studies.

**Methods:**

In order to analyse this relation the literature review of scientific studies on the topic of the relation between the EI and DT was conducted.

**Results:**

The literature sources published in 1994-2023 years were reviewed for this study. 1,87% of studies showed that DT related negatively to general EI. 5,66% showed that some facets of DT related positively to some facets of EI, while 2,83% showed negative relation. 2,83% demonstrated that psychopathy (P) related positively to general EI, 13,21% reported negative relation, 0,94% showed no relation, 2,83% demonstrated mixed relation. 5,66% reported that some facets of P related positively to some facets of EI, while 38,68% discovered negative relation. 14,15% demonstrated that narcissism (N) related both positively and negatively to general EI, 4,72% observed no relation. 16,98% found that some facets of N related positively to some facets of EI, 21,7% showed negative relation. 5,66% demonstrated that M related positively to general EI, 7,55% reported negative relation. 10,38% found that some facets of M related negatively to some facets of EI.

**Conclusions:**

Among reviewed papers most of them reported that some facets of DT related positively to some facets of EI, with P mostly relating negatively to general EI and some of its facets, N mostly relating positively to some facets of EI and M mostly relating negatively to general EI and some of its facets. Found and known limitations of mentioned studies included: sample population being too heterogenous, sample population including criminal offenders only, size of sample population being too small, use of self-report measures, lack of unified approach to measure EI, possibility of comorbidity influencing EI, possibility of individual’s secondary gain influencing EI measure, non-exclusion of individuals with mental disorders from general population. This highlights the need to take mentioned limitations into account in order to acquire accurate data in further research on the topic.

**Disclosure of Interest:**

None Declared

